# IRF8 is crucial for the nicotine withdrawal-induced hyperalgesia in mice

**DOI:** 10.1515/tnsci-2020-0139

**Published:** 2020-09-09

**Authors:** Lina Guo, Yang Zhang, Jinping Wang, Yingying Qi, Zongwang Zhang

**Affiliations:** Department of Anesthesiology, Liaocheng People’s Hospital, Cheeloo College of Medicine, Shandong University, Liaocheng, 252000, Shandong, China; Department of Anesthesiology, Liaocheng People’s Hospital, No. 67 of Dongchang West Road, Liaocheng, 252000, Shandong, China

**Keywords:** nicotine withdrawal, thermal hyperalgesia, IRF8, microglia, P2X4, BDNF

## Abstract

**Background:**

Interferon regulatory factor 8 (IRF8) is involved in the pathogenesis of neuropathic pain. However, whether and how IRF8 can regulate the nicotine withdrawal (NTW)-induced hyperalgesia has not been clarified.

**Methods:**

C57BL/6 mice were randomized and injected subcutaneously with saline (Control) or nicotine (3 mg/kg) three times per day for 7 consecutive days, followed by injection with mecamylamine to induce NTW. Their paw withdrawal latencies (PWLs) were measured, and the relative levels of IRF8 expression in the spinal cord tissues were determined longitudinally by western blot. The numbers of IRF8+ cells in the spinal cord tissues were examined. In addition, the NTW mice were randomized and infused intrathecally with vehicle saline (NS), control lentivirus or lentivirus for the expression of IRF8-specific shRNA for three days. Their PWLs, microglia activation, IRF8 and P2X4R and BDNF expression in the spinal cord tissues were determined.

**Results:**

In comparison with the Control mice, the NTW significantly decreased the PWLs but increased the relative levels of IRF8 expression and the numbers of IRF8+ cells in the spinal cord tissues of mice. IRF8-silencing significantly mitigated the NTW-decreased PWLs and attenuated the NTW-enhanced microglia activation and P2X4R and BDNF expression in the spinal cord tissues of mice.

**Conclusions:**

Spinal IRF8 is crucial for the NTW-induced hyperalgesia by enhancing microglia activation and spinal P2X4R and BDNF expression in mice. The IRF8/P2X4R/BDNF axis may be potential therapeutic targets for postoperative pain of smokers.

## 1 Introduction

Tobacco use is a large and growing global public health burden. Tobacco use is the leading cause of preventable diseases, disability and death, and causes death of many million people worldwide, according to the World Health Organization [1]. It is well known that cigarette smoking is an independent risk factor for the development of cancer, cardiovascular and respiratory diseases [1]. Although tobacco contains many substances, nicotine is a major cause of tobacco dependence [2]. Long-term heaving smoking can lead to nicotine addiction, and when those heaving smokers undergo surgery, they have to withdraw nicotine exposure in the perioperative period, leading to nicotine withdrawal (NTW) syndrome [3,4]. Sudden NTW can reduce pain threshold, and patients with NTW require higher doses of opioids for postsurgical analgesia, which can positively feed back and enhance future smoking [5,6,7]. However, the molecular mechanisms underlying the pathogenesis of NTW-induced hyperalgesia have not been clarified.

Microglia activation in the spinal cord tissues is associated with increased sensitivity to chronic pain and can alter the expression of many factors that contribute to hyperalgesia [8,9]. It is notable that interferon regulatory factor 8 (IRF8), a key nuclear transcription factor, is constitutively expressed by resident microglia and is crucial for the action and motility of microglia [10,11,12]. IRF8 can induce the expression of inflammatory cytokines and other molecules, contributing to the pathogenesis of neuropathic pain, and IRF8 deficiency alters the expression profiles in microglia following different external stimuli. Actually, upregulated IRF8 expression in spinal microglia is associated with the development of tactile allodynia in rodents [13]. However, the role of IRF8 in the NTW-induced hyperalgesia remains unclear. Furthermore, IRF8 can, through IRF5, enhance the expression of P2X purinoceptor 4 (P2X4R), which can promote the P38MAPK activation and the release of brain-derived neurotrophic factor (BDNF) from microglia [14]. The released BDNF can bind to its receptor of TrkB to induce hyperexcitability of microglia, promoting the occurrence and maintenance of neuropathic pain [15]. Our previous studies have shown that aberrant activation of spinal microglia and the P2X4R/BDNF signaling is critical for the development of NTW-induced hyperalgesia in rats [16,17,18]. However, it is unclear whether similar mechanisms can also occur in mice with NTW-induced hyperalgesia.

In this study, we established a mouse model of NTW-induced thermal hyperalgesia and explored the dynamic expression of IRF8 in the spinal cord tissues of mice. Subsequently, we tested the impact of IRF8-silencing by intrathecal injection with lentivirus for the expression of IRF8-specific siRNA after NTW on thermal hyperalgesia and microglia activation, and P2X4R and BDNF expression in the spinal cord tissues of mice.

## 2 Materials and methods

### 2.1 Animals

Male C57BL/6 mice (6–8 weeks old) were purchased from Pengyue Animal Experimental Center (Jinan, China, certificate number SCXK, Shandong 20190003). The mice were housed in a specific pathogen-free facility of Liaocheng Medicine Inspecting Institute (Shandong, China) with a cycle of 12 h light/dark, 25 ± 1°C and free access to food and water *ad libitum*. The experimental protocols were approved by the Institutional Animal Care and Use Committee of the Liaocheng People’s Hospital (Shandong, China) and conducted according to the “Guidelines for the Care and Use of Laboratory Animals” by the National Institutes of Health. All efforts were made to minimize animal suffering and to reduce the number of mice used.


**Ethical approval:** The research related to animals use has been complied with all the relevant national regulations and institutional policies for the care and use of animals. The research project basically conformed to international medical ethics documents and relevant Chinese laws, regulations and ethics requirements. The researchers had guaranteed that they abided by the principles stated by the World Medical Association and respected the ethical recommendations made by the ethics committee on this project. Ethical approval has been granted by Medical Ethics Committee of Liaocheng People’s Hospital affiliated to Shandong University, China (approval number: 2017053).

### 2.2 Lentivirus construction

Lentiviruses for the expression of control shRNA (NC-LV, 5′-TTCTCCGAACGTGTCACGT-3′) or IRF8-specific shRNA (IRF8-RNAi-LV, 5′-ATCCGAGAGCTGCAGCAATTC-3′, NM_008320) were generated by Shanghai Genechem (Shanghai, China). Both IRF8-RNAi-LV and NC-LV were pseudocoated with vesicular stomatitis virus G glycoprotein, which has been demonstrated to target and transduce glial cells effectively [19]. The generated IRF8-RNAi-LV and NC-LV had 2 × 10^9^ TU/mL and 8 × 10^8^TU/mL, respectively.

### 2.3 Model of NTW, treatment and collection of samples

We established a mouse model of NTW, as described previously [17] with minor modifications. Mice were injected subcutaneously with vehicle saline (Control) or 3 mg/kg nicotine (nicotine hydrogen tartrate salt, [−]-1-methyl-2[3-pyridyl]pyrrolidine [Sigma, St. Louis, USA]) in 0.9% normal saline] for three times per day (7 am, 3 pm, 11 pm) for 7 consecutive days. One hour after the last injection on the 7th day, the mice were injected subcutaneously with 1 mg/kg mecamylamine (Sigma) to trigger NTW. The Control and NTW groups (*n* = 20 per group) of mice were tested for their behaviors between in 8 am—and 10 am every day for 1, 4, 7, 10 and 14 days post NTW. After each behavioral assessment, three mice from each group were sacrificed, and their L_4–6_ spinal cords were dissected for western blot. The remaining five mice in each group were sacrificed after day 7 behavioral assessment, and their L_4–6_ spinal cord tissues were dissected for immunohistochemistry.

1 day after injection with mecamylamine, another 36 NTW mice were randomized and infused intrathecally with vehicle saline, NC-LV or IRF8-RNAi-LV at 8 am every day for three consecutive days [20]. Briefly, individual mice were anesthetized and inserted with a 30-gauge microinjection needle connected to a 10 µL Hamilton microsyringe into L4 or L5 spinous space at a 20-degree angle above the vertebrae column. When the need entered the subarachnoid space, the mice were infused with 5 µL saline (NS); the same volume saline containing 4 × 10^6^ TU NC-LV or IRF8-RNAi-LV, respectively. Their behavioral assessments were performed between 8 am and 10 am every day for 7 consecutive days post the last intrathecal infusion (*n* = 12 per group). Subsequently, the mice were sacrificed, and their L_4–6_ spinal cord tissues were dissected for immunohistochemistry (*n* = 5 per group), western blot and quantitative RT-PCR (*n* = 7 per group).

### 2.4 Measurement of thermal hyperalgesia

Individual mice were tested for their thermal hyperalgesia using the BME-410C automatic thermal stimulus apparatus (Medical Science Biomedical Engineering Research, China), as a previous description [21]. Briefly, individual mice were acclimated on the hind paw plantar surface for 15 min and stimulated with heat using a radiant thermal stimulus (a 12 V/10 W halogen lamp with a stimulating light area of <20 mm^2^ and timing accuracy of 10 ms) below the hind paw plantar surface. Each paw was tested for three times with an interval of 5 min, and individual paws were tested for a maximum of 20 s. The paw withdrawal latencies (PWLs) were recorded, and the mean values of PWLs were further analyzed.

### 2.5 Immunohistochemistry

After each behavioral assessment, the mice were anesthetized with pentobarbital sodium (50 mg/kg, intraperitoneal) and perfused transcardially with cold phosphate buffered saline (PBS, 0.1 M, pH 7.4), followed by ice-cold 4% paraformaldehyde in PBS. The lumbar spinal cords (L4–L6 segments) were immediately dissected and post-fixed in 4% paraformaldehyde at 4°C overnight, followed by paraffin-embedding. The tissue sections (−6 µm) were dewaxed, rehydrated and subjected to antigen retrieval. The sections were blocked with 5% bovine serum albumin and incubated with primary antibodies against IRF8 or Iba1 (rabbit anti-IRF8, orb86775, 1:50, Biorbyt, England; rabbit anti-Iba1, ab178847 1:800, Abcam, USA) at 4°C overnight. After being washed, the sections were incubated with biotinylated goat anti-rabbit IgG and SABC (Beyotime, Nanjing, Jiangsu, China), followed by reaction with DAB and counterstained with hematoxylin. The stained signals were captured under a light microscope (Olympus) and semiquantitatively analyzed. The number of IRF8+ cells and the solidity of Iba-1+ microglial cells in five fields of each sample were counted in a blinded manner. The stained intensity was analyzed by ImageJ (NIH, USA) and the solidity of microglia was analyzed, as previous description [22]. In general, the more ramified thinner microglia, the bigger convex area is, thus yielding a smaller solidity index as resting microglia.

### 2.6 Quantitative RT-PCR

The dissected fresh spinal cord tissues were frozen in liquid nitrogen and stored at −80°C until analysis. Total RNA was extracted from each spinal cord sample using the Trizol (Takara, Dalian, China) and reverse-transcribed into cDNA using a Reverse Transcription Kit (Takara). The relative levels of target gene to the control GAPDH mRNA transcripts were determined in triplicate by quantitative RT-PCR using a SYBR Premix Ex TaqTMII (Takara) and specific primers (Sangon Biotech) in an ABI 7500 (Applied Biosystems, USA). The data were analyzed by 2^−ΔΔCt^. The sequences of primers were sense 5′-TGTTCGTGAAGCGGCTGTGC-3′ and antisense 5′-GCTCTCGGATGAACTGGTTGGTG-3′ for IRF8; sense 5′-CTCATCCTGGCTTACGTCATT-3′ and antisense 5′-GAATCCAAGCTGAGAAGTGTTG-3′ for P2RX4; sense: 5′-CCCATGAAAGAAGTAAACGTCC-3′ and antisense 5′-CCCATGAAAGAAGTAAACGTCC-3′ for BDNF; sense 5′-TGTTCGTGAAGCGGCTGTGC-3′ and antisense 5′-GCTCTCGGATGAACTGGTTGGTG-3′ for GAPDH.

### 2.7 Western blot

**Figure 1:**
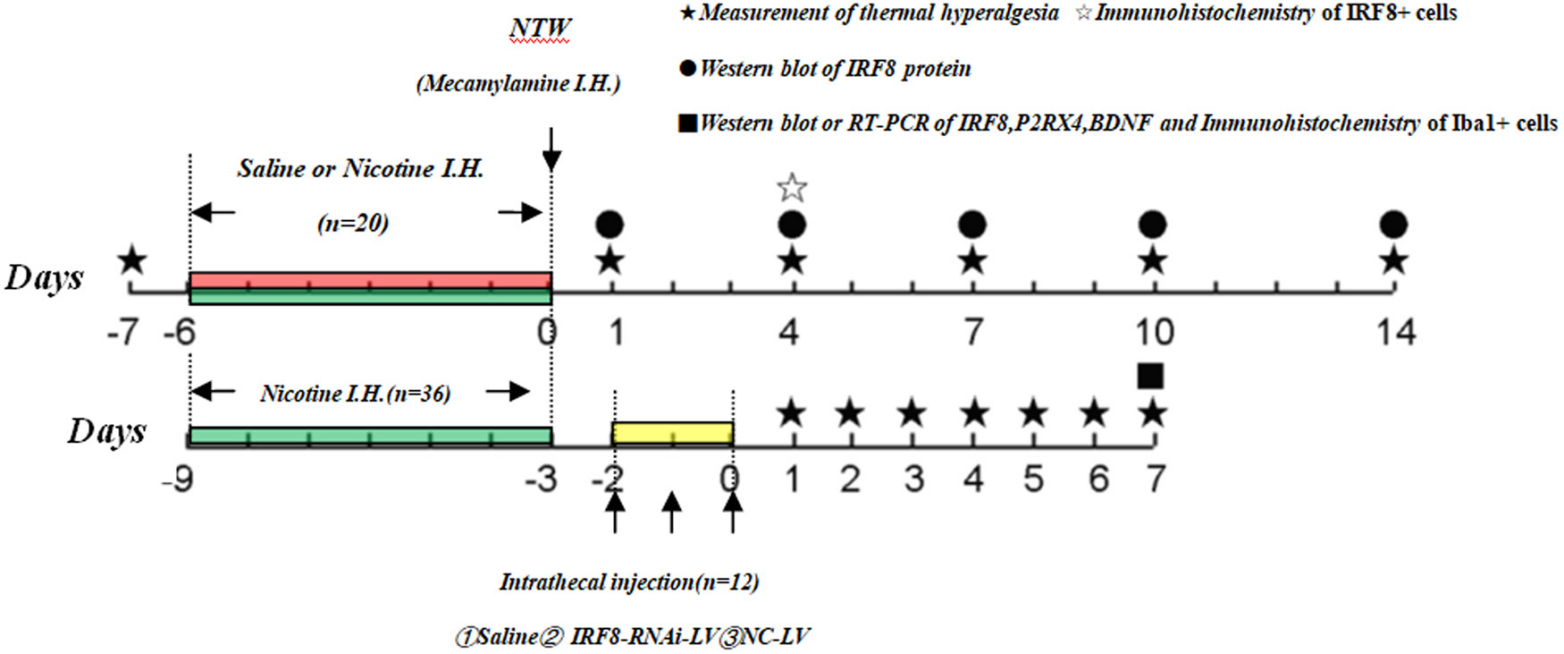
Experimental design and timelines. C57BL/6 mice were randomized and injected subcutaneously with saline or nicotine per day for 7 consecutive days, followed by injection with mecamylamine to induce NTW. Their thermal hyperalgesia were measured and the expression of IRF8 in the spinal cord tissues was determined. In addition, the NTW mice were randomized and infused intrathecally with vehicle saline (NS), control lentivirus (NC-LV), or lentivirus for the expression of IRF8-specific shRNA (IRF8-RNAi-LV) for three days. Their PWLs, microglia activation, IRF8, P2X4R and BDNF expression in the spinal cord tissues were determined.

The frozen spinal cord tissue samples were homogenized in ice-cold lysis buffer containing a cocktail of protease inhibitors and phosphatase inhibitors (Sigma) and centrifuged. After measuring the protein concentrations in the lysate supernatants using BCA Protein Assay Kit (Beyotime Biotechnology), the lysates (15 µg/lane) were resolved by sodium dodecyl sulfate-polyacrylamide gel electrophoresis on 10% gels and transferred onto polyvinylidene difluoride membranes (Millipore, Billerica, MA, USA). The membranes were blocked with 5% nonfat dry milk in TBST and incubated overnight at 4°C with rabbit anti-IRF8 (LS-C335605, 1:600, Lifespan, USA), rabbit anti-P2RX4 (LS-C334869,1:800, Lifespan), rabbit anti-BDNF (28205-1-AP, 1:800, Proteintech, China) or mouse anti-β-actin (1:1,000, AA128, Beyotime). After being washed, the bound antibodies were detected with goat antirabbit or goat antimouse IgG/HRP secondary antibodies (1:1,000, Beyotime) and visualized with enhanced chemiluminescent reagents. The relative levels of target to β-actin protein expression were determined by densitometric analysis using ImageJ (NIH, USA) (Figure 1).

### 2.8 Statistical analysis

Data are expressed as mean ± SD and graphed using Prism 6.0 (GraphPad, La Jolla, CA). The repeated measurements for PWL with multiple time points in each group were analyzed by two-way ANOVA and *post-hoc* Bonferroni’s test. Other quantitative data among the groups were analyzed by one-way ANOVA and *post-hoc* LSD test or Student’s *t*-test. All statistical analyses were performed using SPSS 16.0 statistical software. A *p*-value of <0.05 was considered statistically significant.

## 3 Results

### 3.1 NTW decreases PWLs and increases IRF8 expression in the spinal cord tissues of mice

**Figure 2:**
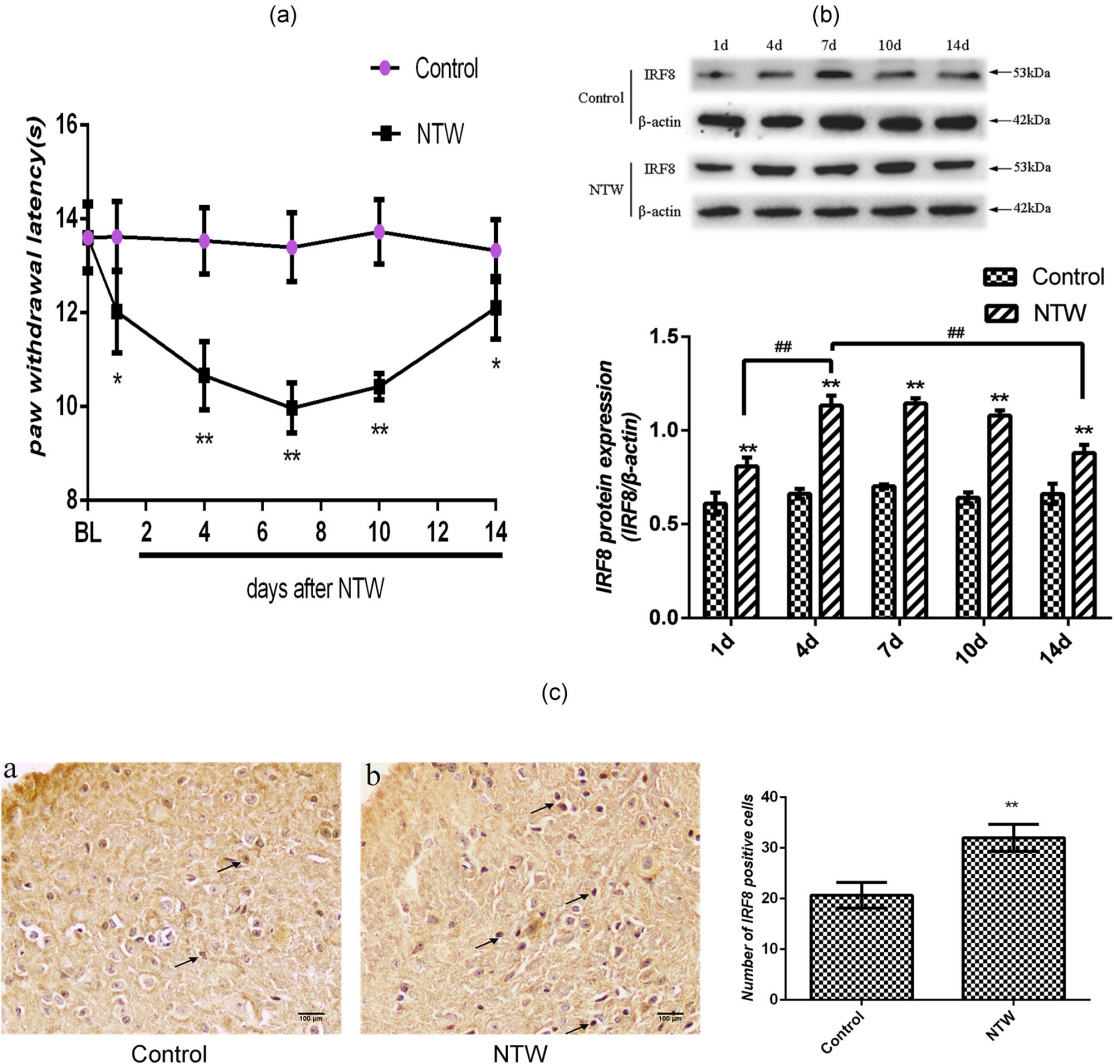
NTW decreases PWLs and increases IRF8 expression in the spinal cord of mice. C57BL/6 mice were randomized and injected with saline as the Control or induced for NTW (*n* = 20 per group). Their thermal hyperalgesia was assessed longitudinally by PWLs after NTW. Some mice (*n* = 3 per group) were sacrificed, and their spinal cord tissues were dissected and tested for the expression of IRF8 by western blot. Some mice (*n* = 7 per group) from each group were sacrificed after behavioral test on day 7 post NTW, and the numbers of IRF8+ microglia in the spinal cord tissues were determined by immunohistochemistry. Data are representative images (magnification × 200) or expressed as the mean ± SD of each group from three separate experiments. (a) The PWLs. (b) Western blot analysis of IRF8 expression. (c) Immunohistochemistry analysis of IRF8+ cells in the spinal cord tissues. *P < 0.05, **P < 0.01 versus the control group. ^*##*^ *P* < 0.01 versus the day 4 time point.

NTW has been associated with a decrease in thermal pain threshold [16,17,18]. To explore the molecular mechanisms underlying the effect of NTW, a mouse model of NTW was established. Examination of these mice indicated that there was no significant difference in the baseline of PWLs in the hind limb between the Control and NTW groups of mice (Figure 2a). In comparison with that in the Control, the PWLs were significantly decreased in the NTW group at 1 day post NTW (*P* < 0.05) and were the lowest in the NTW mice at 7 days post NTW (*P* < 0.01), followed by a gradual raise (Figure 2a). However, the PWLs in the NTW group at 14 days post NTW remained significantly lower than that in the Control (*P* < 0.05). These results clearly indicated that NTW significantly decreased thermal pain thresholds in mice, consistent with our previous studies [16,17,18].

Because IRF8 is related to microglia activation and pain sensitivity [13,14], we began to explore the potential mechanisms underlying the pathogenesis of NTW-related hyperalgesia by testing the relative levels of IRF8 expression in the spinal cord tissues of mice. Western blot analysis revealed that the relative levels of IRF8 expression in the spinal cord tissues of the NTW group were significantly upregulated on day 1, 4, 7, 10, or 14 post NTW, compared with that in the Control mice (*P* < 0.05 or *P* < 0.01, Figure 2b). Interestingly, the highest levels of IRF8 expression appeared on days 4 and 7 post NTW in mice. Consistently, immunohistochemistry indicated that the numbers of IRF8+ cells in the spinal cord tissues on day 7 post NTW were significantly greater than that in the Control mice (*P* < 0.01, Figure 2c). The levels of IRF8 expression in the spinal cord tissues were negatively associated with the PWLs in mice, suggesting that increased IRF8 expression may contribute to decreased thermal PWLs in the NTW group of mice.

### 3.2 IRF8-silencing attenuates thermal hyperalgesia in the NTW mice

**Figure 3:**
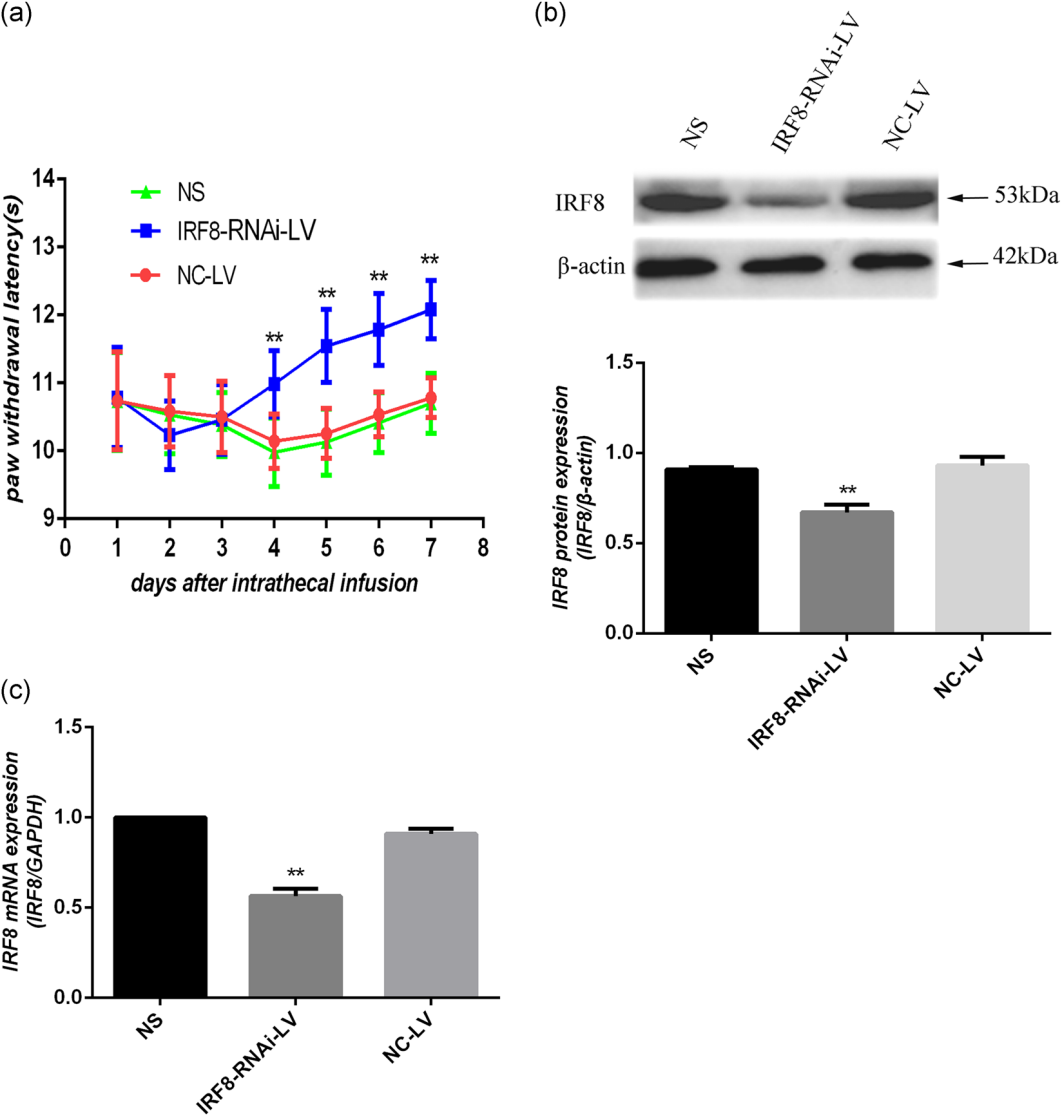
Intrathecal administration with lentivirus (IRF8-RNAi-LV) for IRF8-specific shRNA expression, but not the control NC-LV, attenuates the NTW-induced hyperalgesia in mice. One day after NTW, the mice were randomized and infused intrathecally with vehicle saline, NC-LV or IRF8-RNAi-LV every day for three consecutive days. Their behavioral assessments were performed daily for 7 consecutive days post the last intrathecal infusion (*n* = 12 per group). Subsequently, the mice were sacrificed and their L_4–6_ spinal cord tissues were dissected, and the relative levels of IRF8 expression were determined by western blot and quantitative RT-PCR (*n* = 7 per group). Data are representative images (magnification × 200) or expressed as mean ± SD of each group from three separate experiments. (a) The PWLs. (b) Western blot analysis of IRF8 expression in the spinal cord tissues. (c) Quantitative RT-PCR analysis of IRF8 mRNA transcripts. **P < 0.01 versus the NS group.

To further confirm the role of IRF8 in NTW-induced hyperalgesia, we tested whether knockdown of IRF8 expression in the spinal cord tissues could mitigate the NTW-induced hyperalgesia in mice. First, we generated lentiviruses for the expression of IRF8-specific shRNA (IRF8-RNAi-LV) or control RNA (NC-LV). The NTW mice were randomized and infused intrathecally with vehicle saline (NS), control NC-LV or IRF8-RNAi-LV every day for three consecutive days, and their PWLs were tested daily up to 7 days post the last virus infusion. There were similar levels of PWLs between the NS and NC-LV groups of mice, indicating that infusion with NC-LV did not affect the NTW-induced hyperalgesia in mice (Figure 3a). Compared with the NC-LV or NS groups, intrathecal infusion with IRF8-RNAi-LV significantly increased PWLs in the NTW mice on day 4 post the last virus infusion (*P* < 0.01, Figure 3a) and continually prolonged up to 7 days post virus infusion in the NTW mice. In addition, we found that the relative levels of IRF8 expression in the spinal cord tissues of the IRF8-RNAi-LV group of mice were significantly reduced by about 27% compared with other two groups of mice (*P* < 0.01, Figure 3b). Similarly, IRF8-silencing also significantly decreased the relative levels of IRF8 mRNA transcripts in the spinal cord tissues of the NTW mice (*P* < 0.01, Figure 3c). Thus, IRF8-silencing mitigated the NTW-induced thermal hyperalgesia in mice.

### 3.3 IRF8-silencing mitigates the NTW-stimulated microglia activation and P2X4R and BDNF expression in the spinal cord tissues of mice

**Figure 4:**
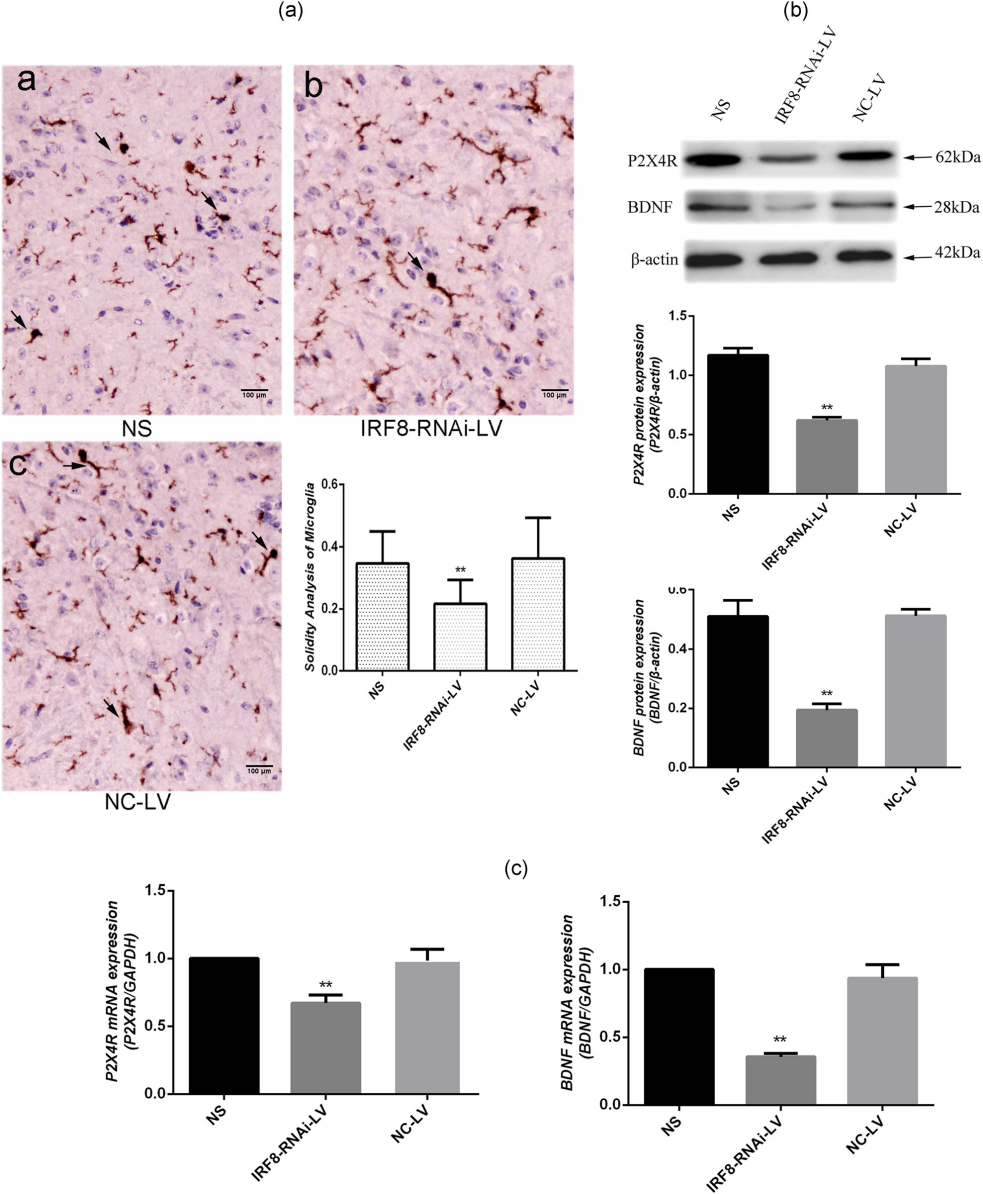
IRF8-silencing mitigates the NTW-enhanced microglia activation, P2X4R and BDNF expression in the spinal cord tissues of mice. On day 7 post the last virus infusion, the different groups of mice were sacrificed and their spinal cord tissues were dissected. (a) Immunohistochemistry analysis of microglia using anti-Iba1 and quantification of the solidity of microglia in the spinal cord tissues. (b) Western blot analysis of P2X4R and BDNF expression. (c) Quantitative RT-PCR analysis of P2X4R and BDNF mRNA transcripts in the spinal cord tissues of mice. Data are representative images (magnification ×200) or expressed as mean ± SD of each group (*n* = 5 per group) from three separate experiments. **P < 0.01 versus the NC-LV group.

Our previous study has shown that NTW increases microglial cell activation and upregulates P2X4R and BDNF expression in the lumbar spinal cord of rats [18]. To understand the mechanisms underlying the action of IRF8-silencing in attenuating thermal hyperalgesia in mice, we tested whether IRF8-silencing could modulate the NTW-stimulated microglial cell activation, P2X4R and BDNF expression in mice. Immunohistochemistry analysis revealed that there were many microglial cells with a bigger body and shorter and thicker processes, the hallmarks of activated microglia in the spinal cord tissues of the NS and NC-LV groups of mice while many microglial cells displayed smaller body with longer and thinner processes, and the characteristics of resting microglia in the spinal cord tissues of the IRF8-RNAi-LV group of mice (Figure 4a). Quantitative analysis indicated that the solidity index of microglia in the spinal cord tissues of the IRF8-RNAi-LV group of mice was significantly less than that of the NS and NC-LV groups of mice (*P* < 0.01, Figure 4a).

Western blot analysis revealed that the relative level of P2X4R and BDNF protein expression in the spinal cord tissues of the IRF8-RNAi-LV group was significantly reduced by 50–62%, compared with that in the NS and NC-LV groups of mice (*P* < 0.01, Figure 4b). A similar pattern was detected for P2X4R and BDNF mRNA transcripts among groups of mice (Figure 4c). Collectively, such data demonstrated that decreased IRF8 expression significantly mitigated the NTW-stimulated microglial cell activation and P2X4R and BDNF expression in the spinal cord tissues of mice.

## 4 Discussion

In the present study, we found that sudden nicotine deprivation decreased the pain threshold, but increased IRF8 expression, accompanied by increased numbers of IRF8+ cells in the spinal cord tissues of mice. The increase in the expression of IRF8 began on day 1 post NTW and lasted for at least for 14 days, consistent with the decreasing trend of thermal PWLs. Our previous study has shown that the PWLs display a V-shape throughout the 7-day tests and reach the peak on day 4 post NTW in rats. However, the PWLs reached the peak on day 4 and maintained at the peak until day 10 post NTW, followed by decline to the first-day level on day 14 post NTW. The difference may stem from different experimental animals and testing instruments. Furthermore, IRF8-silencing by intrathecal injection with lentivirus for expression of IRF8-specific shRNA significantly mitigated the NTW-decreased PWLs by reducing microglia activation and P2X4R and BDNF expression in the spinal cord tissues of mice. These findings extended previous observations that upregulated IRF8 expression contributed to the NTW-induced thermal hyperalgesia in rats [16,17,18], supporting the notion that the IRF8/P2X4R/BDNF signaling is important for the NTW-induced hyperalgesia.

The worldwide ban of indoor smoking requires long-term heaving smokers to stop nicotine exposure suddenly when they are hospitalized. NTW may have a neurophysiologic effect linked to increased pain sensitivity [23]; however, the precise effect of NTW on pain sensitivity remains abstinence. While some studies indicate that abstinence of sustained smoking can decrease pain sensitivity, other studies believe that NTW may increase pain sensitivity, particularly during the early stages of NTW [6,7,24,25]. A previous study has suggested that the NTW-induced hyperalgesia may be mediated by the activity imbalance of β2*-neuronal nicotinic ACh receptors (nAChRs) [26]. Furthermore, NTW can cause anxiety, depression and insomnia, and decrease the activation of the hypothalamus–pituitary–adrenal axis, which increase pain sensitivity [27]. In addition, NTW can upregulate the expression of corticotropin-releasing factor and its receptor 1, which are important mediators of hyperalgesia in the central amygdala [28]. In this study, we found that NTW increased microglia activation, IRF8 and P2X4R and BDNF expression in the spinal cord tissues. Moreover, IRF8-silencing mitigated the NTW-induced hyperalgesia by decreasing microglia activation and P2X4R and BBDNF expression in the spinal cords of mice. Such data indicated the NTW-enhanced microglia and IRF8/P2X4R/BDNF signal activation contributed to the NTW-induced hyperalgesia in mice. Hence, our findings may provide new insights into the molecular mechanisms underlying the pathogenesis of NTW-related hyperalgesia.

Aberrant activation of microglia is important for evoking neuropathic pain, cancer-related pain and morphine tolerance [8,9]. Our previous studies and those of others have shown that NTW induces microglia activation in the central nervous system (CNS) and is closely related to hyperalgesia [16,29,30]. It is notable that IRF8 is expressed in the nucleus of microglia and is critical for microglia activation. In this study, we observed that the upregulated IRF8 levels were negatively associated with the levels of PWLs in mice following NTW, extending previous observations in neuropathic pain models [31]. More importantly, we found that IRF8-silencing significantly mitigated the NTW-induced hyperalgesia and reduced microglia activation and P2X4R and BDNF expression in the spinal cord tissues of mice. These findings demonstrated lentivirus-mediated IRF8-specific shRNA effectively silenced IRF8 expression in the spinal cells and the importance of IRF8 in the pathogenesis of NTW-induced hyperalgesia. Actually, the strategy of lentivirus-mediated gene expression has been widely used in the pain research field because this strategy can induce highly efficient transgene expression with a high safety profile in the CNS [32]. Theoretically, the IRF8/P2X4R/BDNF signaling may be new therapeutic targets for the intervention of neuropathic pain in those heaving smokers when they are hospitalized.

In this study, we found that NTW upregulated P2X4R and BDNF expression while IRF8-silencing mitigated the NTW and upregulated their expression in the spinal cord tissues of mice, accompanied by increased PWLs. These data were consistent with our previous observations [16,17,18], indicating that the IRF8/P2X4R/BDNF signaling was crucial for the NTW-induced hyperalgesia and other types of neuropathic pain [14,33]. Previous studies have shown that the fibronectin/integrin signaling, chemokine 21 and chemokine (C–C) ligand 2 (CCL2) [34,35], the IRF8/IRF5 signaling and transcription factor PU.1 are important for P2X4R expression in microglia in the CNS [14]. It is possible that NTW may enhance IRF8 expression, which, through IRF5 together with PU.1, induces P2X4R expression that promotes the P38MAPK activation and the release of BDNF from microglia, leading to microglia hyperexcitability and neuropathic pain. Therefore, these findings may uncover the pathogenic factors contributing to the NTW-induced hyperalgesia.

We recognized that this study had several limitations. First, we centered on the spinal cord, but not other nociception-related brain areas, including the prefrontal cortex, hippocampus and amygdala, which may underestimate the outcome of NTW in mice. Second, this study focused on the IRF8/P2X4R/BDNF signaling but did not investigate other pathways, which may miss some outcomes of altered IRF8 expression. Thus, further investigations are warranted to determine the precise mechanisms by which NTW induces hyperalgesia.

In conclusion, our data indicated that NTW induced hyperalgesia and upregulated IRF8 expression in the spinal cord tissues of mice, and IRF8-silencing significantly mitigated the NTW-induced hyperalgesia and decreased the NTW-enhanced microglia activation and P2X4R and BDNF expression in the spinal cord tissues of mice. To the best of our knowledge, this was the first evidence to demonstrate the importance of the IRF8/P2X4R/BDNF signaling in the pathogenesis of NTW-induced hyperalgesia. Therefore, our findings may provide new insights into the pathogenesis of NTW-induced hyperalgesia and uncover new therapeutic targets for the intervention of neuropathic pain, particularly for those heaving smokers.
